# Rotation insensitive implantable wireless power transfer system for medical devices using metamaterial-polarization converter

**DOI:** 10.1038/s41598-024-70591-4

**Published:** 2024-08-24

**Authors:** Tarakeswar Shaw, Bappaditya Mandal, Gopinath Samanta, Thiemo Voigt, Debasis Mitra, Robin Augustine

**Affiliations:** 1 Department of Electrical Engineering, Microwaves in Medical Engineering Group, Division of Solid-State Electronics, Uppsala University, 75121 Uppsala, Sweden; 2 Department of Electronics and Communication Engineering, The Lakshmi Niwas Mittal Institute of Information Technology, Jaipur, 302031 Rajasthan India; 3 Department of Electrical Engineering, Division of Networked Embedded Systems, Uppsala University, 75121 Uppsala, Sweden; 4 Department of Electronics & Telecommunication Engineering, Indian Institute of Engineering Science and Technology, Shibpur, 711103 India

**Keywords:** Engineering, Biomedical engineering, Electrical and electronic engineering

## Abstract

This article introduces an innovative approach for creating a circular polarization (CP) antenna-based rotation-insensitive implantable wireless power transfer (WPT) system for medical devices. The system is constructed to work in the industrial, scientific, and medical (ISM) frequency band of 902–928 MHz. Initially, a flexible, wide-band, and bio-compatible open-ended CP slot antenna is designed within a single-layer human skin tissue model to serve as the receiving (Rx) element. To form the implantable WPT link, a circular patch antenna is also constructed in the free-space to use as a transmitting (Tx) source. Further, a new metamaterial-polarization converter (MTM-PC) structure is developed and incorporated into the proposed system to enhance the power transfer efficiency (PTE). Furthermore, the rotational phenomenon of the Rx implant has been studied to show how the rotation affects the system’s performance. Moreover, a numerical analysis of the specific absorption rate (SAR) is conducted to confirm compliance with safety regulations and prioritize human safety from electromagnetic exposure. Finally, to validate the introduced concept, prototypes of the different elements of the proposed WPT system were fabricated and tested using skin-mimicking gel and porcine tissue. The measured results confirm the feasibility of the introduced approach, exhibiting improved efficiency due to use of the MTM-PC. The amplitude of the transmission coefficient ($|S_{21}|$) has improved by 6.94 dB in the simulation, whereas the enhancement of 7.04 dB and 6.76 dB is obtained from the experimental study due to the integration of MTM-PC. As a result, the PTE of the proposed MTM-PC integrated implantable WPT system is increased significantly compared to the system without MTM-PC.

## Introduction

In the past few years, the design of compact and high-performance wireless power transfer (WPT) systems for biomedical applications has gained tremendous attention within the scientific community^1^. WPT systems give a safe and reliable path to transmit power without any physical contact^2^. In addition, WPT systems eliminate the issue of limited lifetime of batteries used in implantable medical devices (IMDs) and hence save the patients from critical surgery, which is usually required to replace the battery^3,4^. In this context, WPT systems offer the best solution to eliminate the issue of batteries by charging them wirelessly. Henceforth, significant research effort has been conducted to construct high-performance WPT systems to charge IMDs^5,6^.

It is noted from the literature that initially, the research has concentrated on designing a non-radiative resonator-based WPT system for implantable devices^7–10^. However, resonator-based WPT systems are designed to operate in a lower MHz frequency range. Therefore, the size of the receiving (Rx) implants is large, and some designs have 3-D bulky configurations^7–10^, which limits the real-time application of the non-radiative methodology. In addition, the non-radiative-based approach is highly sensitive to misalignment (lateral and angular) and is used for short-range applications^11,12^. However, an efficient magnetic coupling-based implantable WPT system^13^ integrated with negative permeability (MNG) metasurface has been illustrated to eliminate the misalignment issues and improve the performance for biological applications. Moreover, the implementation of a simple, planar, miniaturized implantable WPT system along with better efficiency and transfer distance is highly required to fulfill the recent demand to charge IMDs wirelessly.

To overcome the issue of a resonant-based WPT system, the first breakthrough has been made by Poon et al.^14^ to find out the optimal frequency to obtain enhanced performance in implantable scenarios. It has been explained that to achieve improved performance from the implantable biomedical environment^14^, the operating frequency should be in the Sub-GHz and GHz frequency ranges. Thereafter, notable research has been performed on the design of antenna-based implantable WPT system^15–21^ due to its compact size and directive radiation. Most recently, metamaterial (MTM) integrated radiating antenna-based implantable WPT systems have demonstrated improved efficiency^22–24^. However, most of the previous work concentrated on the implementation of linear-polarized (LP) antenna-based implantable WPT systems^15–24^. Usually, LP-based WPT systems suffer from loss due to multi-path interference and polarization mismatch.

**Fig. 1:**
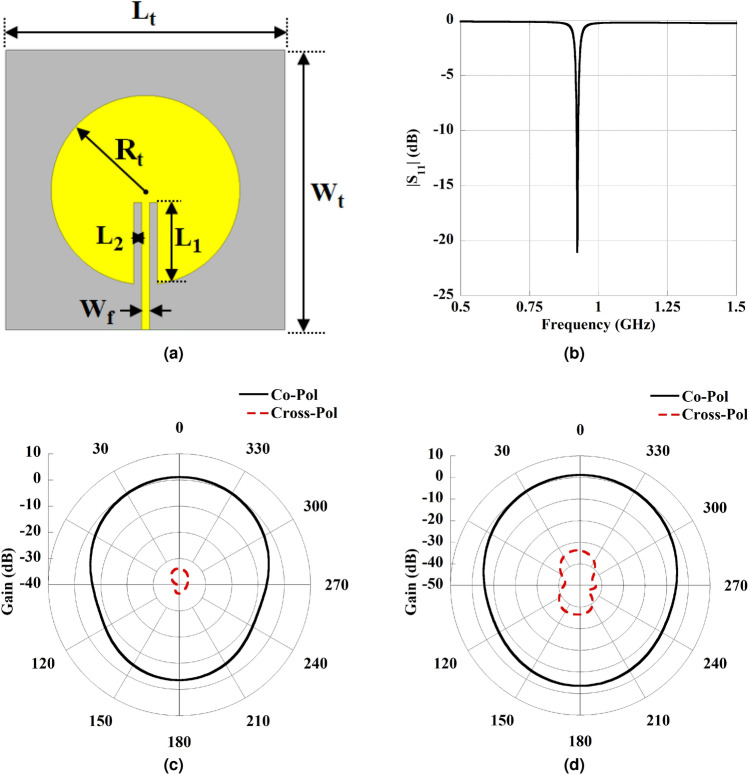
Schematic diagram and parameter of the circular patch antenna. (**a**) Top view with the detailed dimension of patch. where, $L_t=W_t=90$ mm, $R_t=30.4$ mm, $L_1=26.32$ mm, $L_2=1.4$ mm, and $W_f=1.57$ mm. (**b**) Property of the return loss of the Tx patch antenna. The 2D plot of the radiation pattern at 920 MHz, (**c**) E-plane, and (**d**) H-plane.

Hence, to eliminate the issues of the LP-based approach, some works constructed circular polarization (CP) based implantable WPT systems^25–28^. The CP antenna based approach allow more arbitrary positioning of the implanted device relative to the external power source. It reduces the need for precise alignment between the Tx and Rx antennas, making the system more robust and user-friendly. Moreover, the CP antenna base systems are less susceptible to multipath interference, which can occur due to signal reflections within the body. A compact CP implant antenna has been constructed using stub loading to design an implantable WPT system^25^. Meanwhile, a miniaturized CP patch antenna operating at 915 MHz has been designed for implantable biomedical applications^26^. Further, to show the mismatch effect between LP-Tx and CP-Rx, two Tx antennas^25,26^ with different polarization’s have been considered. The reported studies^25,26^ show that the maximum power transmission is obtained when the Tx and Rx implant antennas are circularly polarized. Moreover, a zero-index MTM-based solution has constructed an efficient CP-antenna-based WPT system for IMD^27^. Recently, an efficiency enhancement methodology by utilizing a parasitic patch for a CP antenna-based implantable WPT system^28^ has been discussed. In most of the reported LP/CP-based works, superstrate dielectric material is kept on the implantable Rx antenna to overcome direct contact among the human body and radiating elements. However, placement of the additional superstrate increases the implant Rx element’s thickness along with the uncertainty of bio-compatibility. Notably, very few research articles aim to improve the PTE of the CP antenna-based system despite its necessity to model a high-performance implantable system. Therefore, designing a planar, compact, rotation-insensitive, bio-compatible WPT system over a flexible dielectric with enhanced efficiency is highly preferred for IMD applications.

**Fig. 2:**
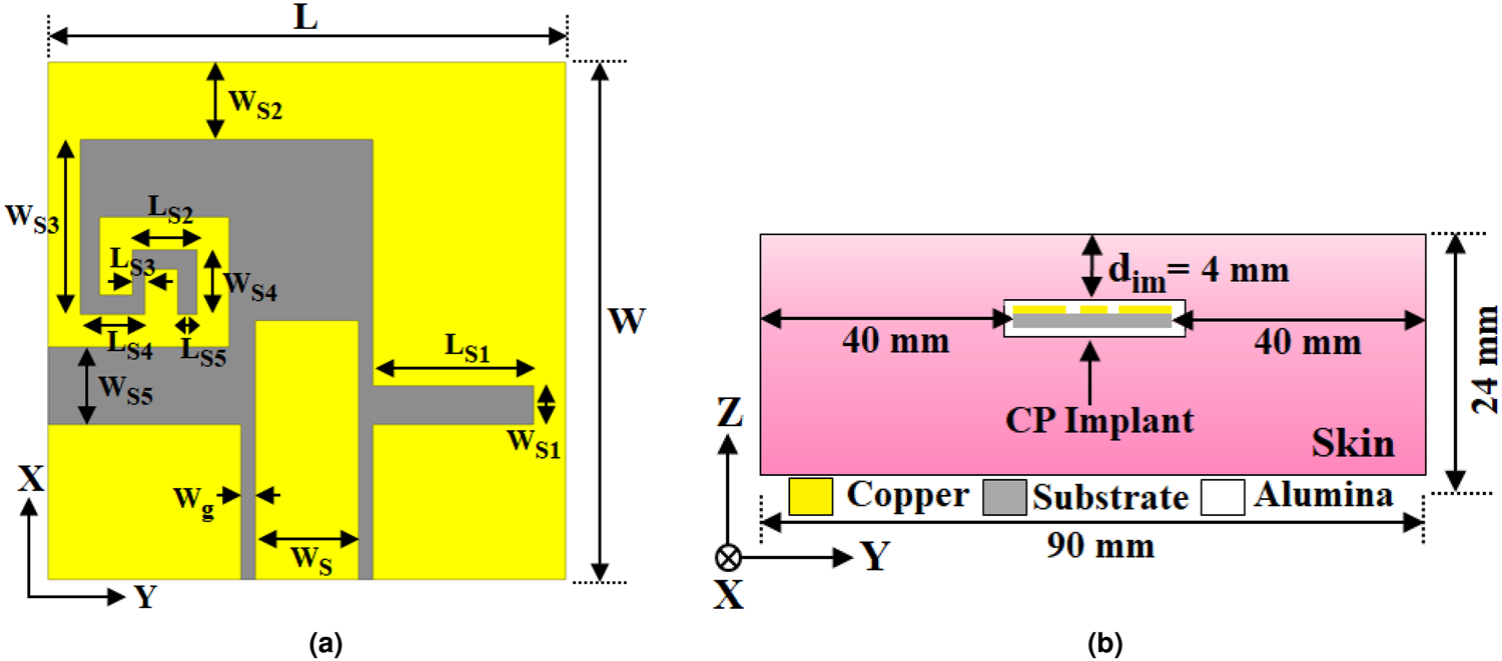
Geometry of the CPW-fed implantable Rx open-ended slot antenna. (**a**) Top view. where $W=10$ mm, $L= 10$ mm, $W_S = 2$ mm, $W_g = 0.27$ mm, $W_{S1} = 0.75$ mm, $W_{S2} =1.5$ mm, $W_{S3} = 3.375$ mm, $W_{S4} = 1.25$ mm, $W_{S5} = 1.5$ mm, $L_{S1} = 3.105$ mm, $L_{S2} = 1.25$ mm, $L_{S3} = 0.25$ mm, $L_{S4} = 1.25$ mm, and $L_{S5} = 0.375$ mm. (**b**) Side view of the implanted CP antenna in single-layer skin tissue model.

**Fig. 3:**
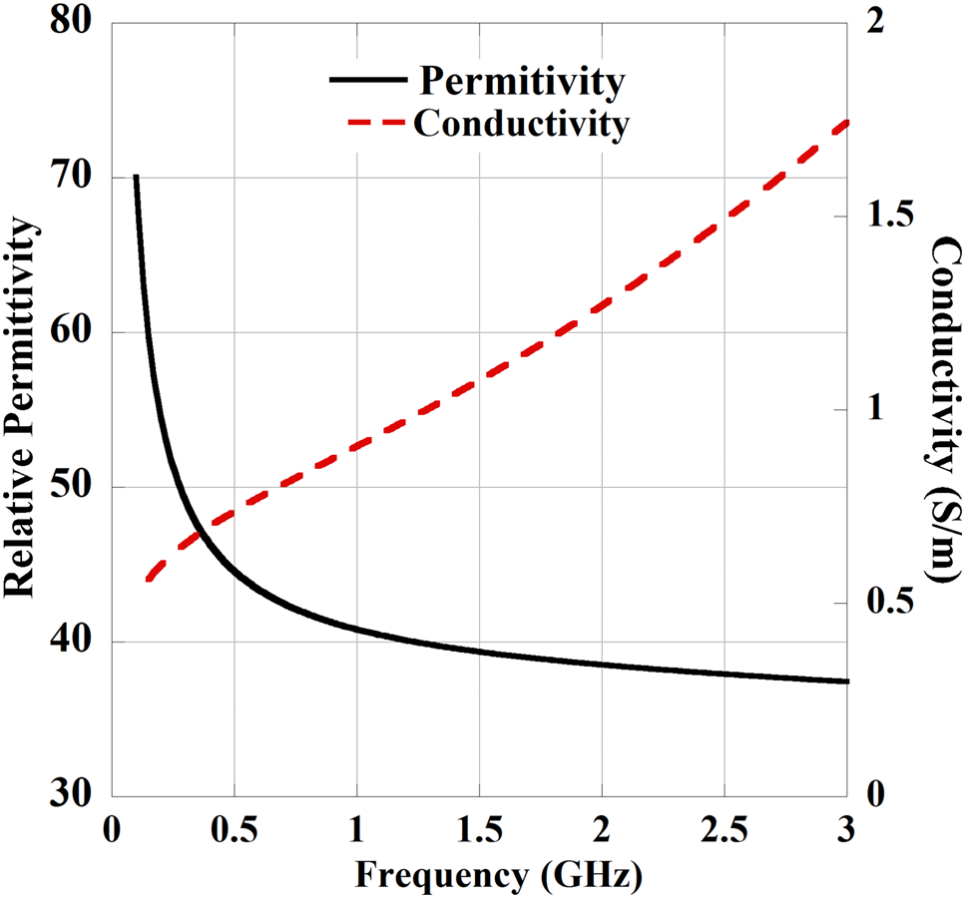
Characteristics of the skin tissue with the change in frequency.

In this article, to meet these requirements, a rotation-insensitive CP antenna-based, bio-compatible, miniaturized implant WPT system on a flexible platform has been designed with enhanced efficiency for the IMDs. The introduced system is constructed to operate in the frequency band of 902–928 MHz. The selected ISM frequency band provides better penetration, reduced absorption in human tissue, and increased power transfer distance. A flexible dielectric material is used for the construction of the CP-receiving implant antenna in the human skin tissue model. An alumina ($Al_2O_3$) coating layer is deposited over the implant antenna to eliminate the high-profile issue. Also, a simple patch antenna is constructed in free-space outside the human body where it works as a Tx element and provides energy to the Rx implant. Herein, an LP-Tx antenna has been used instead of two different LP and CP-Tx antennas as considered in reported works^25,26^ to show the polarization mismatch issue between Tx and Rx implants. Further, to enhance the performance of the introduced WPT system, a high-refractive-index (HRI) metamaterial-polarization converter (MTM-PC) has been used. To the best of the author’s knowledge, the first-time MTM-PC based approach has been presented to enhance the performance of the CP implantable WPT system. The HRI-based PC is utilized to convert the radiated LP energy from the Tx antenna to CP, which eliminates the mismatch issue among the Tx and implant Rx antennas. In addition, the HRI property of the MTM-PC simultaneously improves the gain of the Tx antenna on the broadside and focuses the electromagnetic (EM) energy on the Rx implant, leading to the enhancement of PTE for the system. To show the rotation-insensitive behavior, the Rx antenna has been rotated from $0^{\circ }$ to $360^{\circ }$ with the interval of $45^{\circ }$ on the XY plane. From the analysis of rotational characteristics, nearly stable performance has been attained by the proposed system. A detail-specific absorption rate (SAR) analysis has been conducted regarding safety. Finally, to establish the proposed concept, the Tx, Rx, and an array of $4\times 4$ unit cells of the MTM-PC have been fabricated, and measurements were performed. The experimental findings validate the feasibility of the introduced approach by increasing the coupling strength among the Tx and Rx elements by more than 6.5 dB. As a result, the efficiency of the proposed system is enhanced from 0.05 to 0.27%.

**Fig. 4:**
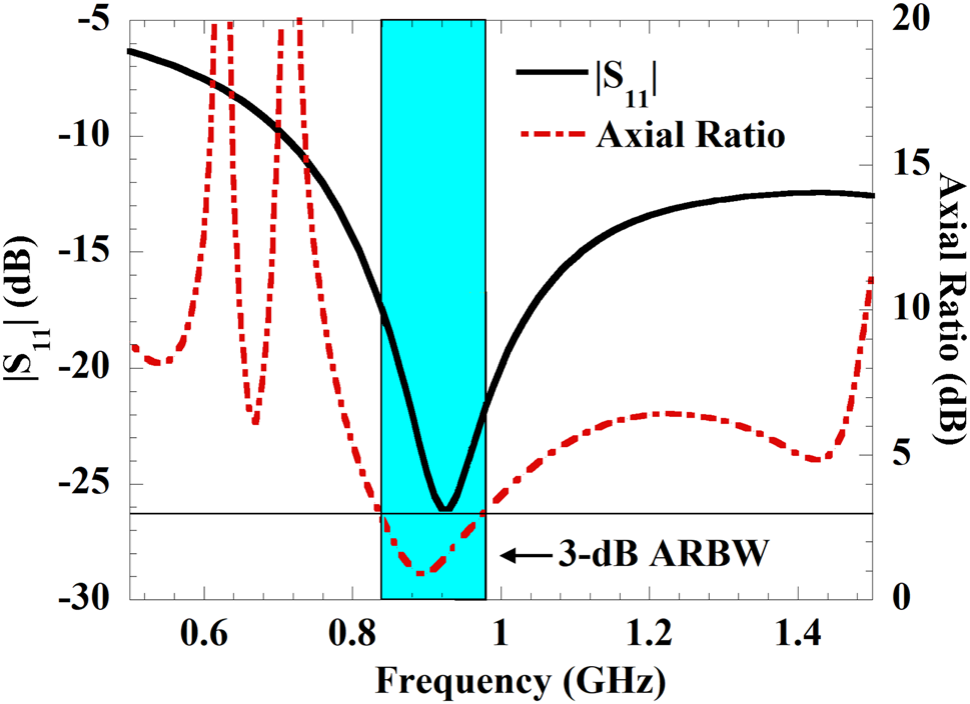
Simulated return loss and axial ratio characteristics of the implantable CP antenna.

**Fig. 5:**
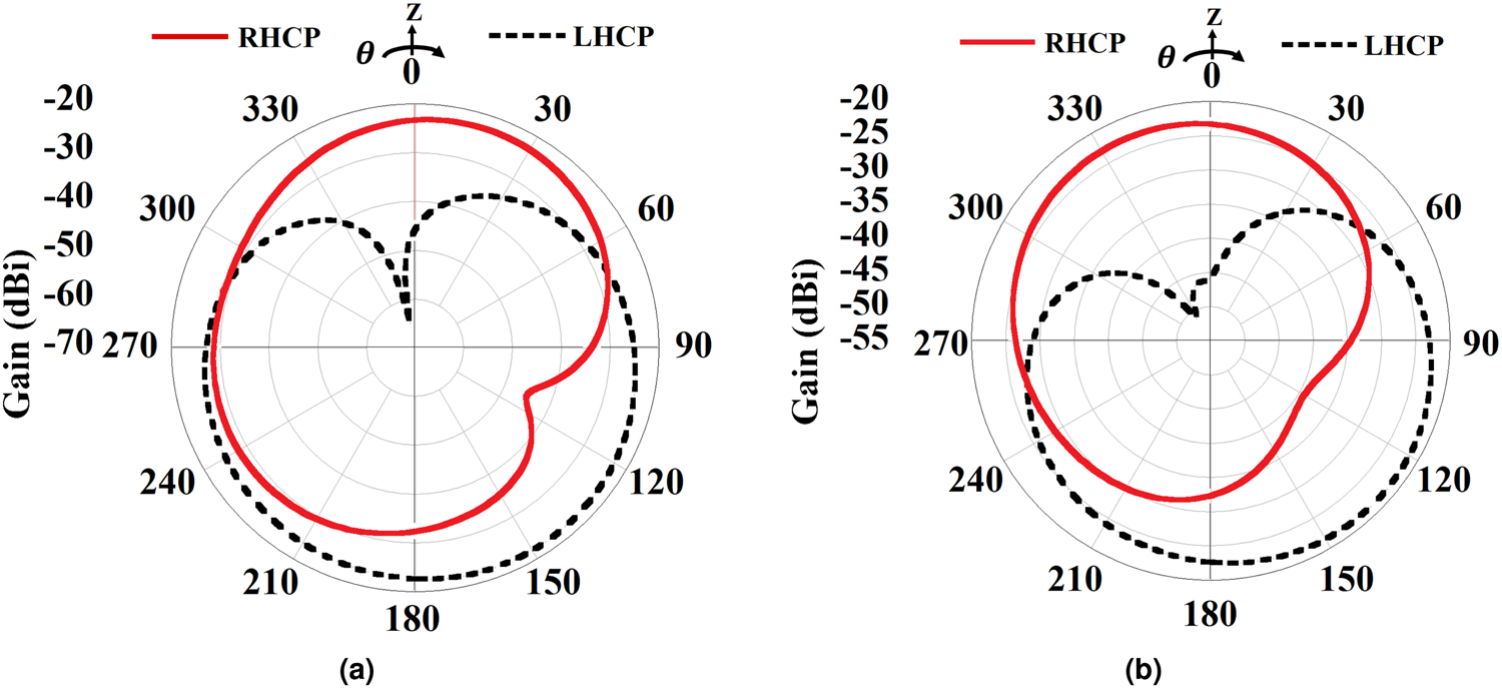
The 2D radiation plot of the implantable Rx slot at 920 MHz. (**a**) xoz-plane, and (**b**) yoz-plane.

## Design and analysis of Tx, Rx implant antenna, and MTM-polarization converter to implement the WPT system

### Construction of the transmitting (Tx) antenna in free-space

This section details the construction of a Tx antenna, which is placed in free-space outside the human body to serve as a source. In biomedical applications, simplicity is the prime concern along with better performance. Therefore, a simple and conventional microstrip-inset feed circular patch antenna is used as a Tx due to its low profile, broadside directive pattern, and can also be easily integrated into the system^23^. The geometric configuration of the Tx antenna is depicted in Fig. 1a. It is constructed on a 1.67 mm thick Rogers RO 3010 substrate with a permittivity of 10.2 and a loss tangent, tan$\delta =0.0035$. Numerical simulations are conducted to refine the patch dimensions, ensuring optimal performance at the desired operating frequency, 920 MHz. The optimized parameters are detailed in Fig. 1. Figure 1b exhibits the simulated return loss property of the patch antenna, while Fig. 1c and d present the 2D radiation phenomenon for the E and H planes of the patch, respectively. Herein, a narrow-band (Fig. 1b) Tx antenna is designed to operate efficiently at a specific resonant frequency. This precise tuning ensures that the maximum amount of power is transferred from the external Tx to the implantable Rx. In addition, the narrow band minimizes the energy losses that can occur when signals are spread over a wide range of frequencies, thus maximizing the power delivered to the implantable device^17,23^. The designed configuration of the Tx patch achieves a gain of 1.09 dB at 920 MHz. All the simulation studies were conducted using Ansys-HFSS high-frequency structure simulator software.

**Fig. 6:**
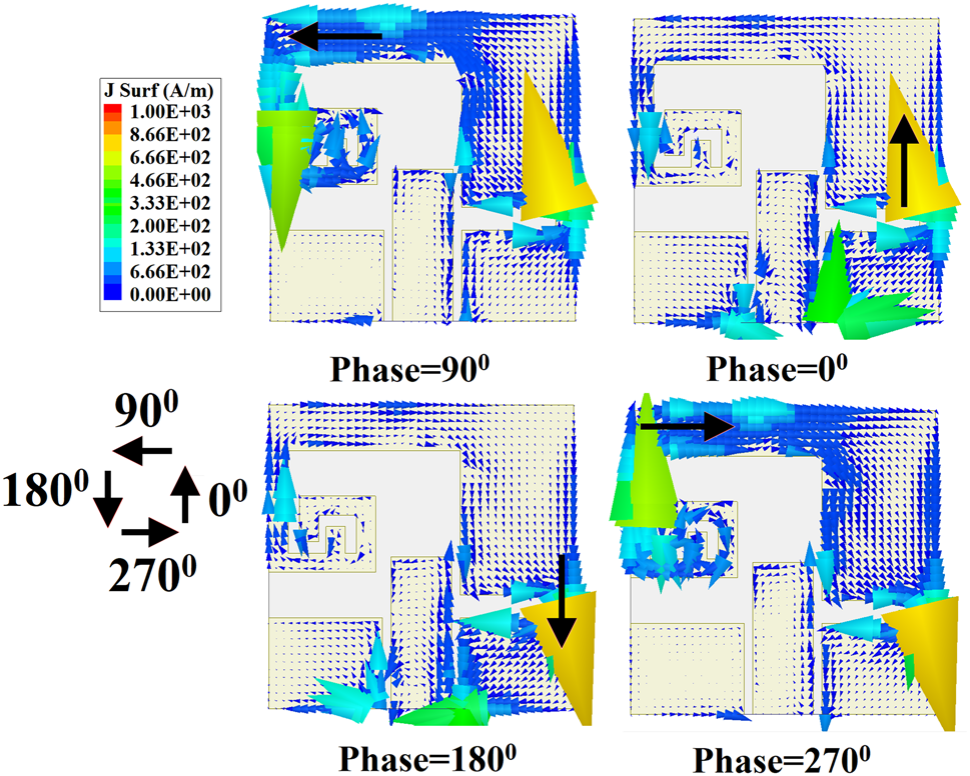
Distribution of surface current on implant slot at 920 MHz for various phase angles.

### Design of the open-ended receiving (Rx) implantable slot antenna

The implantable Rx antenna plays a dominant role in the construction of a high-performance WPT system within the challenging, lossy implant environment. According to the literature^15–28^, high-gain implantable antennas are crucial for optimal system performance. However, the gain is substantially reduced due to the compact size of the antenna and the complex lossy nature of the implant environment. Henceforth, the bio-compatible CPW-fed circularly polarized (CP) implant slot antenna, without the use of vias or shorting pins, is designed as the Rx element for the WPT system. Usually, the human tissues exhibit no magnetic losses, making the radiation of a magnetic source more efficient than an electric one under the same conditions for investigation^29^. Thus, the Rx antenna is designed with a configuration that is minimally influenced by the skin tissue due to its magnetic field-controlled operating region^29^. The schematic configuration of the CPW-fed CP implantable open-ended slot is presented in Fig. 2a. The slot is designed on a flexible square dielectric material with an aperture area of $W \times L$, a thickness of 0.25 mm, a relative permittivity of 10.2, and a loss tangent of 0.0023. For efficient radiation and better performance from the implant antenna, size should be a fraction of the wavelength. At the proposed operating frequency, the dimension of the Rx implant antenna will achieve a good balance between being small enough to fit inside the IMDs and large enough to maintain acceptable performance. Further, the implant antenna is small enough to be placed within the human body without causing discomfort or significant disruption to surrounding tissues. The Rx antenna is implanted at a depth of 4 mm from the surface of the single-layer skin-tissue model with the size of $90 \times 90 \times 24$ $\textrm{mm}^{3}$ shown in Fig. 2b. During simulations, a 0.02 mm thin layer of bio-compatible alumina is applied around the implant slot to prevent direct contact with human tissue^30^. The frequency-dependent permittivity ${\hat{\varepsilon }(\omega )}$ and conductivity $\sigma (\omega )$ of the skin tissue are determined using multiple Cole-Cole dispersion expressions detailed in^31^ and also presented by the Eqs. (1), (2).

1 $$\begin{aligned} {\hat{\varepsilon }(\omega )}= & \varepsilon _\infty +\sum _{n}\frac{\Delta \varepsilon _{n}}{1+(j\omega \tau _{n})^{(1-\alpha _{n})}}+\frac{\sigma _{i}}{j\omega \varepsilon _{0}} \end{aligned}$$

2 $$\begin{aligned} \sigma (\omega )= & -\Im [{\hat{\varepsilon }(\omega )}]\omega \varepsilon _0 \end{aligned}$$

Further, the property of the human skin tissue with the change in frequency is illustrated in Fig. 3 as presented in reported works^23,27^. A CPW is used as the feed line for the antenna where the strip width is $W_S$ and the gap spacing is $W_g$. In the slot configuration, the open-end slot and two U-shaped slots introduce the perturbation in the distributions of magnetic current in the direction of x and y. This generates the circularly polarized wave by stimulating two orthogonal modes in the x and y directions with equal amplitude and quadrature phase difference.

**Fig. 7:**
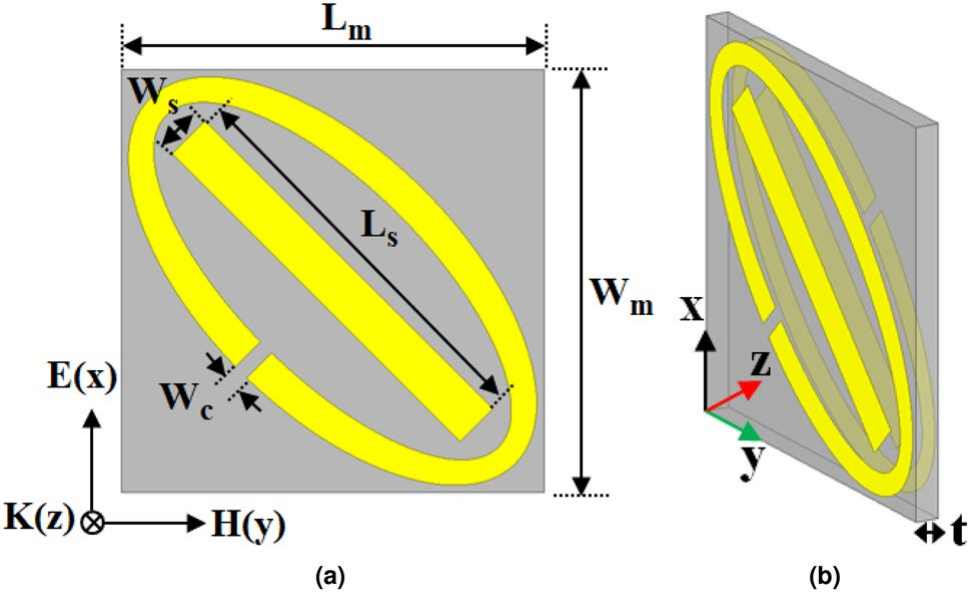
Unit cell structure of the MTM-based polarization converter (**a**) front view, and (**b**) perspective view. Where, $L_m=W_m=27$ mm, $L_s=26$ mm, $W_s=4$ mm, $W_c= 0.2$ mm, and $t =1.67$ mm.

The simulated axial ratio and return loss characteristics of the implant slot are shown in Fig. 4. It can be perceived from the figure that the antenna provides a wide impedance bandwidth (− 10 dB) along with a 3-dB axial ratio bandwidth of 21.87% covering the 902–928 MHz ISM frequency band. The antenna radiation pattern for both xoz and yoz-planes are depicted in Fig. 5. The antenna exhibits right-hand circular polarization (RHCP) radiation phenomena at 920 MHz frequency with a peak gain of − 23.35 dBi and co-cross pol discrimination of 22 dB at the bore sight direction. To understand the CP mechanism, the surface electric current distribution at different phase angles has been captured and plotted in Fig. 6. It is prominent that the electric surface current rotates anticlockwise over the antenna at 920 MHz, which simply implies that the polarization sense of the implant open-ended slot is RHCP in the + Z direction. In the context of CP implantable antenna design, the authors presented the initial study in their most recently reported work^32^.

### Design of the metamaterial-polarization converter (MTM-PC)

Recently, MTMs have gained significant attention due to their unique electromagnetic phenomena like negative permittivity, permeability, and refractive index. These properties make them useful for different applications in radio frequency (RF), microwave, and optical frequency domains^33^. Generally, two-dimensional MTM-PC structures are being used successfully to transform LP patch and slot antennas into CP antenna characteristics^34,35^. This conversion is achieved with improved performance compared to traditional methods. The use of MTM-based CP antennas helps to overcome the challenges associated with designing and achieving better performance than conventional CP antennas. This suggests that the MTM approach simplifies the design process and enhances antenna performance^34,35^.

In this article, an MTM-based LP-to-CP converter is utilized to eliminate the mismatch between the LP-Tx antenna and the CP-Rx antenna. In addition to mismatch elimination between the antennas, the novel HRI property of MTM has also improved the performance of the Tx patch with directive radiation. Consequently, the PTE and the transfer distance of the introduced WPT system have been improved significantly. The unit cell structure of the MTM-PC designed to improve the performance of the introduced implantable system is depicted in Fig. 7. The unit cell is designed with double-sided ellipse-shaped ring resonators along with a rectangular strip line presented in Fig. 7. The two elliptical patterns on top and bottom layers are similar, but rotated with each other by an angle of $90^{\circ }$ around the center z-axis. The rectangular strip line manages the right and left-handedness of the radiated energy from the Tx antenna^35^. By rotating the position of the ellipse and rectangular strip, the antenna’s performance can be optimized for right-hand circular polarization (RHCP) or left-hand circular polarization (LHCP) according to the specific requirements^35,36^. The arrangement depicted in Fig. 7 is considered to achieve broadside radiation of RHCP waves from the Tx patch, aiming to enhance the PTE of the proposed system as the Rx implant antenna also provides RHCP.

**Fig. 8:**
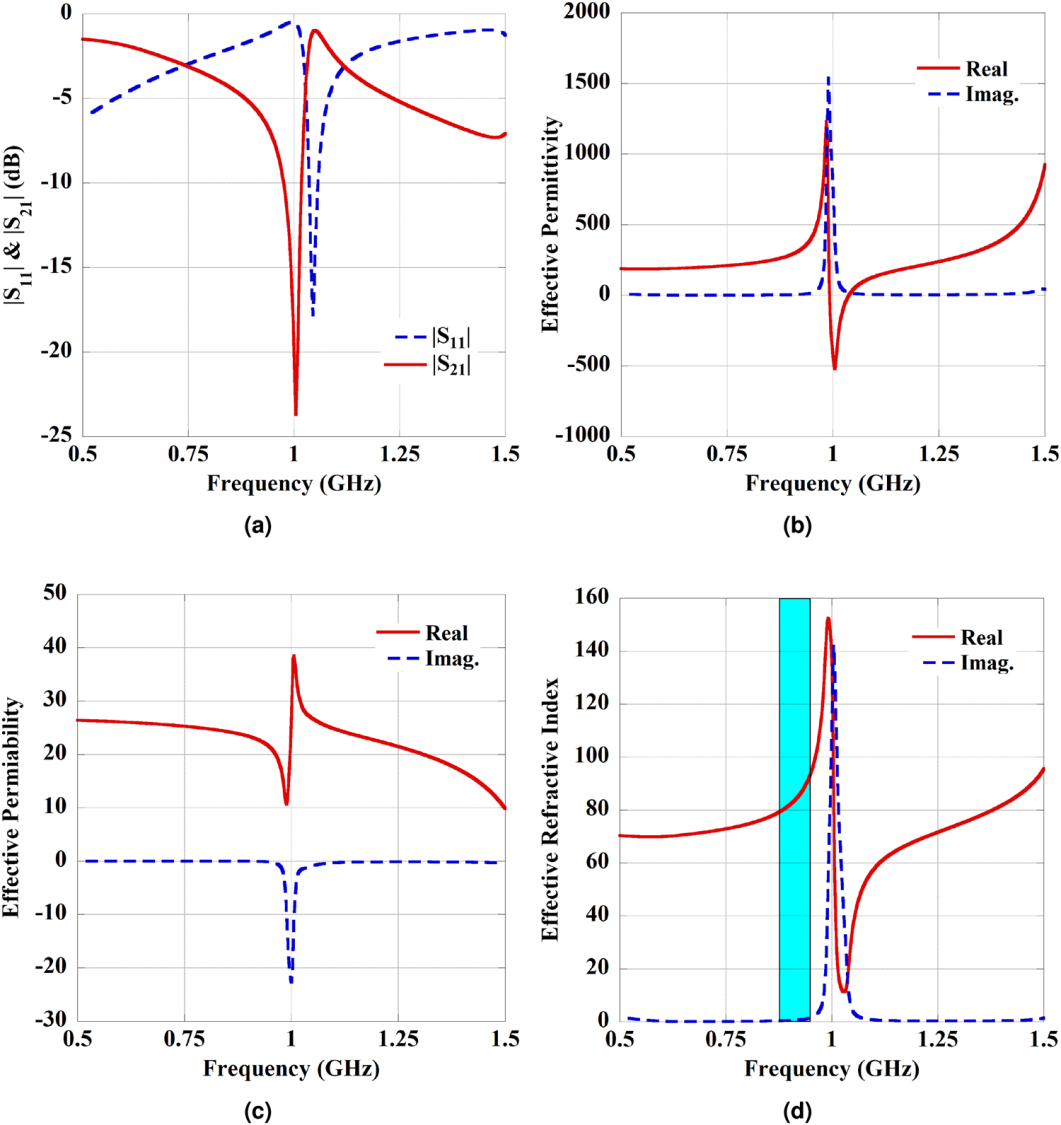
Numerical study of the S-parameter characteristics and extracted effective parameters of the MTM-PC unit cell, (**a**) properties of S-parameter, (**b**) permittivity, (**c**) permeability, and (**d**) refractive index.

**Fig. 9:**
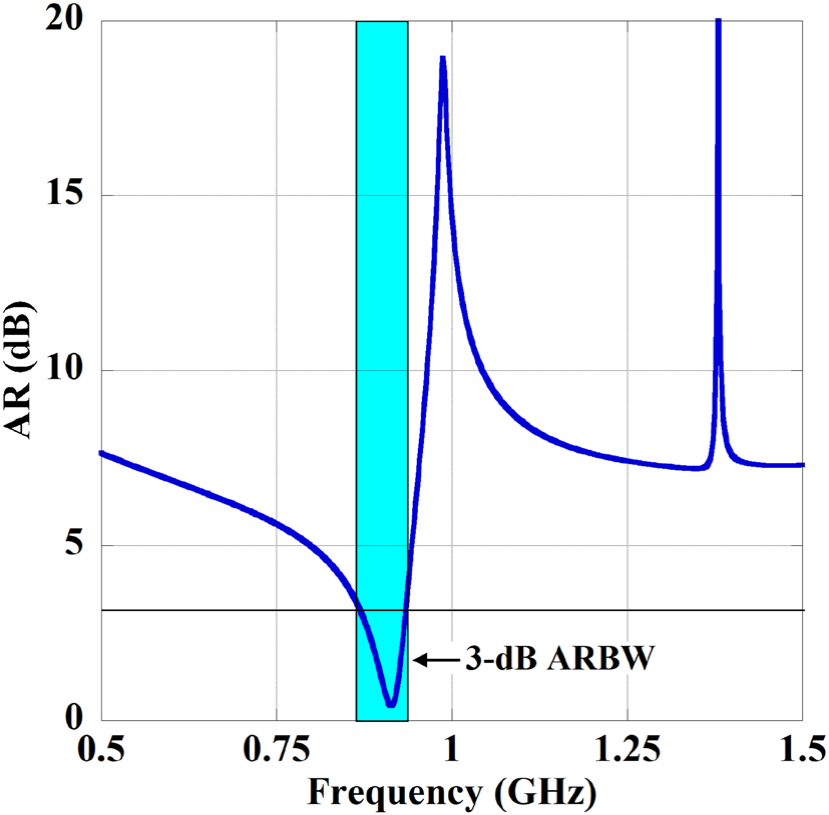
Simulated property of AR for the MTM-PC structure.

**Fig. 10:**
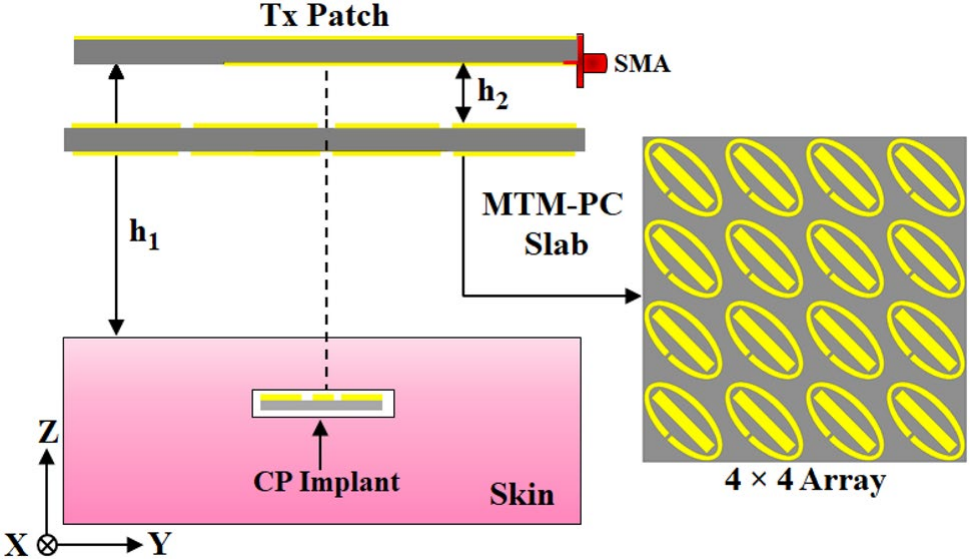
Schematic configuration of the proposed implantable WPT system incorporated with the MTM-PC.

The MTM-PC is designed on the Rogers RO3010 (tm) dielectric substrate with dielectric properties: $\epsilon _r = 10.2$ and tan$\delta =0.0035$. The investigation and extraction of the MTM parameters are achieved by assigning periodic boundary constraints. In Fig. 7a, the alignment of the excited electric field (E), magnetic field (H), and the wave vector (K) is illustrated with respective directions. The numerical simulations are conducted to optimize the dimensions of unit cell, aiming to achieve high refractive index (HRI) and low loss properties along with polarization conversions phenomenon at the required frequency of 920 MHz. The optimized dimensions to attain the desired characteristics from the MTM-PC are given in Fig. 7. The elliptical major axis radius of 16.5 mm and minor radius of 15 mm have been considered to construct the ellipse in the HFSS simulation. The simulated reflection ($|S_{11}|$) and transmission ($|S_{21}|$) coefficients of the MTM structure are illustrated in Fig. 8a. The Kramers-Kronig retrieval method^37^ is employed to extract the effective medium parameters. Figure 8b–d, shows the extracted effective permittivity, permeability, and refractive index of the MTM-PC, respectively. The shaded region in Fig. 8d denotes the specific operating zone aimed to enhance the performance of the WPT system. It can be seen from the figure that the real part of the refractive index is high within this frequency range, while the imaginary part is low, indicating a low-loss behavior in the shaded zone.

Furthermore, another objective of the designed MTM-PC is to control the polarization of an incident wave initially linearly polarized. The designed structure has the capability to convert linear polarization into circular polarization, specifically to RHCP. This characteristic is evident when examining the axial ratio (AR) of the wave that traverses the structure. The AR can be expressed as^36,38^:

3 $$\begin{aligned} AR(\text {dB}) = \text {Mag}\left( 20 \log (AR(\omega ))\right) \end{aligned}$$

where $$AR(\omega )$$ represents:

4 $$\begin{aligned} AR(\omega ) = \frac{T(\omega ) \text { (cross-polar)}}{T(\omega ) \text { (co-polar)}} \end{aligned}$$

and,

5 $$\begin{aligned} T(\omega ) = \text {Mag}(S_{12}) \end{aligned}$$

The AR, defined as the ratio of cross-polarized wave to co-polarized wave, serves as an indicator of CP conversion quality^36,38^. To visualize the AR, Floquet port excitation with master and slave boundaries is also considered on the MTM-PC. The characteristic of the AR obtained from the MTM-PC is presented in Fig. 9. A crucial reference point for PC is usually considered the 3-dB point. If the AR values are below 3 dB, the structure proves effective as a polarization converter. It is observed from Fig. 9 that the unit cell structure exhibits notably large AR bandwidths in the frequency range of 870–930 MHz, which covers the desired ISM band. Moreover, from the parameter extraction and AR analysis of the proposed MTM-PC, it can be noted that the structure provides HRI and low loss properties along with polarization conversion phenomenon in the required frequency regime of 902–928 MHz.

**Fig. 11:**
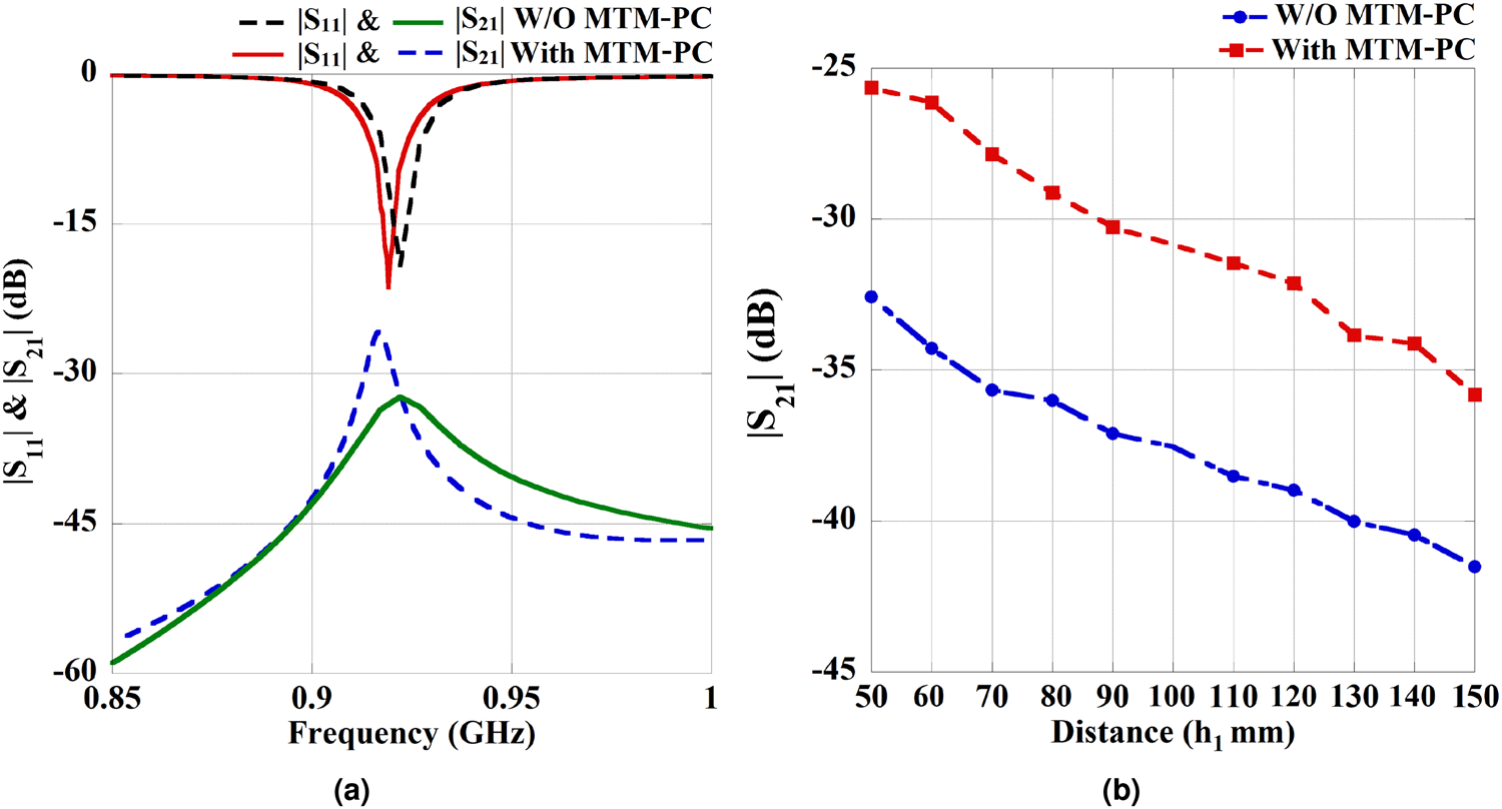
(**a**) The simulated S-parameter characteristics of the WPT system for with and without integration MTM-PC. (**b**) Variation in the amplitude of $|S_{21}|$ with distance ($h_1$).

**Fig. 12:**
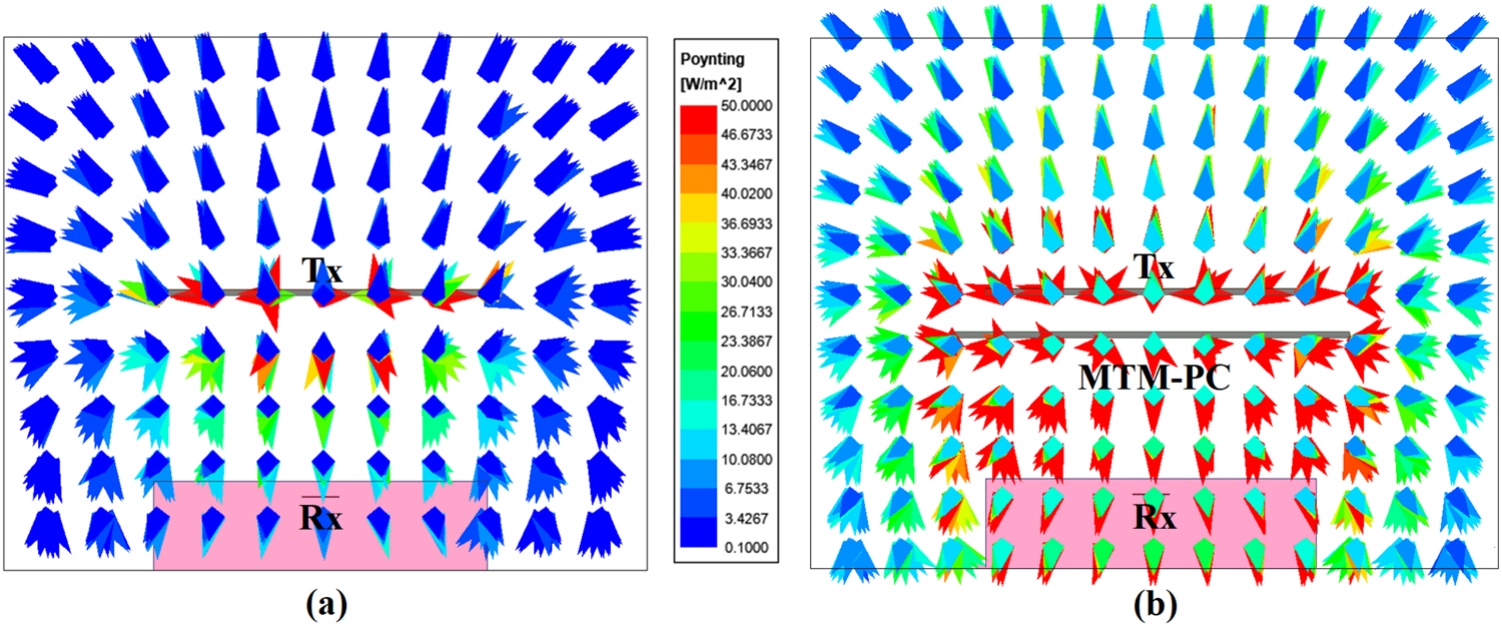
Distribution of the Poynting vector. (**a**) without, and (**b**) with integration of the MTM-PC.

## Proposed MTM-PC integrated implantable WPT system for IMDs

In this section, the simulation setup of the proposed WPT link is illustrated, incorporating the previously designed Tx and Rx antennas along with the MTM-PC discussed in the preceding sections. The configuration setup of the proposed WPT link, including the MTM-PC, designed for IMDs, is represented in Fig. 10. The MTM-PC structure having HRI properties is used to improve the system efficiency and transfer distance. The distance from the Tx patch to the skin surface is denoted as $h_1$, while an MTM-PC slab, comprising a $4\times 4$ unit cell array, is positioned at $h_2$ distance from the Tx patch. The proposed implantable WPT system is designed to operate in the radiative near-field of the Tx patch antenna. In this region, the radiated beams are confined within a specific range, enabling higher transfer distances and reduced sensitivity to misalignment, even with a small Rx element footprint^15,39^. Conversely, in the reactive near-field region, the antenna is dominated by evanescent waves that rapidly diminish with distance^39^. As a result, the reactive near-field is limited to small separation distances between the Tx and Rx elements. Additionally, the system’s performance in this region is highly sensitive to misalignment. Further, the selection of optimal unit cell combinations and precise placement position ($h_2$) is crucial, as the system’s performance is highly dependent on these two factors^13,17,23,27^. To optimize system performance, two steps of optimization have been performed: firstly, optimizing the combination of unit cells on the MTM-PC, and secondly, optimizing the placement distance, $h_2$, from the Tx patch. A series of numerical studies have been carried out to identify the optimal array combination of the unit cells and corresponding placement distance ($h_2$) to enhance system performance. The detailed discussion regarding the optimization process of array combinations and placement distance is illustrated in previous studies^17,23,27^. The numerical studies show that an array of $4\times 4$ cells and the distance of $h_2 = 5$ mm provides the most significant improvement in the transmission coefficient ($S_{21}$), significantly enhancing efficiency. By the use of the high refractive index MTM property, the effective aperture of the Tx patch is increased, which enhances the directive radiation from the Tx patch with improved gain towards the Rx implant^17,40^. In addition, the PC characteristics of the MTM mitigate the issue of the mismatch due to polarization among the Tx and Rx antennas. Consequently, the efficiency of the presented MTM-PC loaded WPT system is enhanced significantly. The efficiency of the WPT is quantified through the power transfer efficiency ($\eta$), described by the squared magnitude of the transmission coefficient^13,17^ as, $\eta = |S_{21}|^{2}$.

**Fig. 13:**
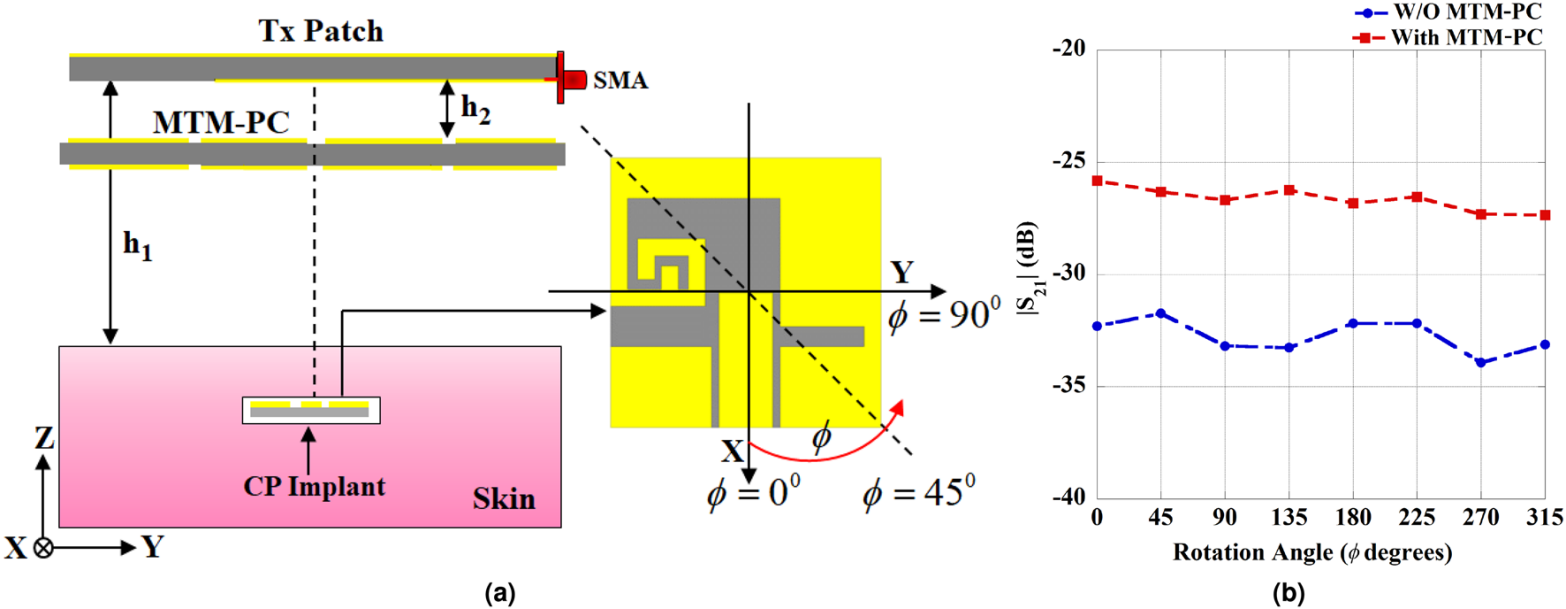
(**a**) Geometrical setup for investigating the rotational influence of the Rx implantable antenna. (**b**) The effect of rotation on the magnitude of transmission coefficient.

**Fig. 14:**
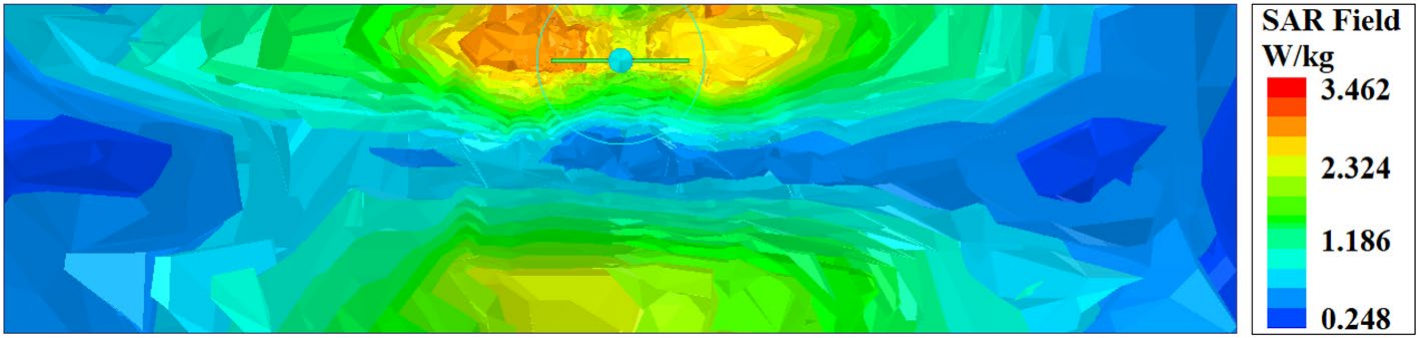
The simulated 1-g average SAR distribution on single-layer skin tissue.

Furthermore, a comparative analysis of the simulated S-parameters is conducted for the proposed WPT system, both with and without inclusion of the MTM-PC. The variations in S-parameter characteristics at $h_1 = 50$ mm and $h_2 = 5$ mm with $4\times 4$ array of unit cells are shown in Fig. 11a. It is evident from Fig. 11a that the utilization of the MTM-PC significantly enhances the coupling strength of the transmission coefficient near 919 MHz, leading to a significant improvement in efficiency. The impact of transfer distance ($h_1$) on system performance is analyzed with MTM-PC fixing at 5 mm away from the Tx antenna. Fig. 11b represents the obtained characteristics of the transmission coefficient ($|S_{21}|$) with and without integration of the MTM-PC. As usual, the transmission strength tends to decrease with an increase in transfer distance. However, the inclusion of MTM-PC has significantly enhanced the coupling strength of $S_{21}$, even as the distance varies, resulting in a considerable improvement in PTE. A noteworthy 6.94 dB increment in coupling strength is observed due to the use of MTM-PC at a transfer distance ($h_1$) of 50 mm.

Moreover, to present the effect of MTM-PC on the proposed system, the distribution of the Poynting vector is illustrated in Fig. 12 without and with presence of the MTM-PC, respectively. The distribution suggests that incorporating MTM-PC results in the concentration of radiated energy from Tx antenna over the Rx slot with higher intensity than the system without MTM-PC. This concentration of EM energy improves the system’s efficiency.

## Rotational effect and safety analysis of the introduced WPT system

This section covers the rotational effect, safety considerations, and numerical study of the specific absorption rate (SAR) for the proposed implantable WPT system. In LP antenna-based WPT systems^15–19,22,23^, it is necessary to orient them in-phase to achieve improved PTE. Therefore, this study employs a CP implant antenna to construct the WPT system to ensure performance that is insensitive to rotation. The schematic configuration to present the rotational characteristics among the Tx and Rx antennas is illustrated in Fig. 13a. In this setup, the implanted slot undergoes rotation in the plane of XY, varying the rotational angle ($\phi$) from $0^{\circ }$ to $360^{\circ }$ at intervals of $45^{\circ }$, while the positions of the source patch and MTM-PC remain stable. The variation in maximum amplitude of $|S_{21}|$ with and without the MTM-PC for various rotational angles is also presented in Fig. 13b. The figure illustrates a minor variation in the peak coupling strength of $|S_{21}|$ attributed to the use of the CP-based approach. In addition, the inclusion of the MTM-PC results in a significant enhancement in the amplitude of $|S_{21}|$, leading to a substantial improvement in the overall PTE of the system.

**Fig. 15:**
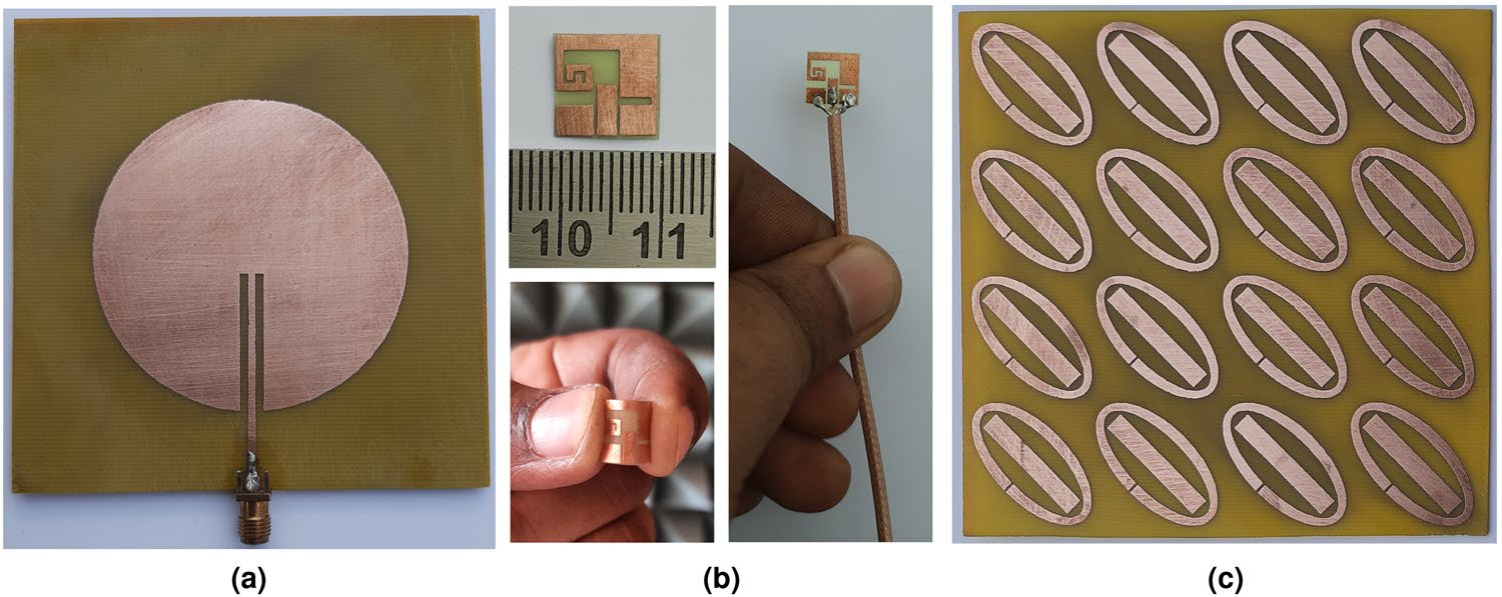
Pictures of the fabricated prototypes. (**a**) Tx patch, (**b**) Rx implant antenna, flexibility, connected coaxial cable, and (**c**) array of $4\times 4$ MTM-PC.

**Fig. 16:**
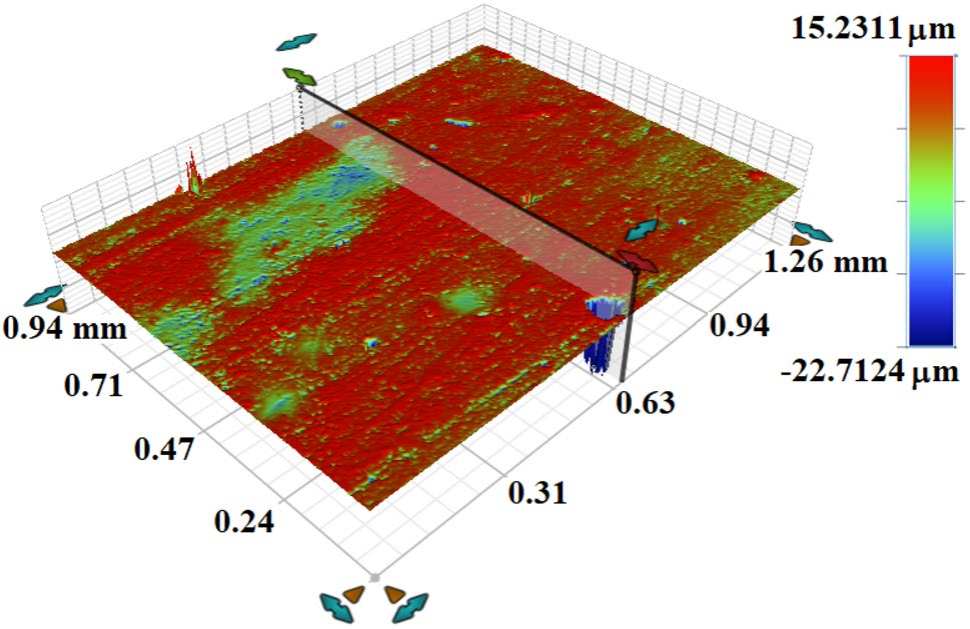
The 3D optical perspective view of the alumina coating around the implant slot.

The prime objective during the design of an implantable WPT system is the safety of the human from EM exposure. To ensure the bio-compatibility of the implant CP slot, a coating layer of alumina is applied over the implant slot instead of using a superstrate dielectric that would increase the implant’s profile^13,17,18,24,25,28^. Typically, the SAR value is used to assess the suitability of the designed implantable system for practical application. According to the guidelines of the Federal Communications Commission (FCC)^41^, the peak Tx output energy impinges over the Rx implant is 1 W (30 dBm) at the ISM bands. Additionally, to ensure patient safety, the SAR value should be maintained within a specific range, as per the standard of IEEE C95.1-1999. For safety reasons, the 1 g average SAR in human tissue of a cubic model must be less than or equal to 1.6 W/kg^42^.

Herein, the simulation study of SAR is performed in the HFSS software to ensure the real-time utilization of the introduced CP-based implant WPT system for biomedical applications. The investigation of SAR was performed on the MTM-PC-loaded implantable WPT system. The distribution of the simulated average SAR over 1 g of the skin tissue model is presented in Fig. 14, revealing an optimal SAR value of 3.462 W/kg. At the time of simulation, the Tx patch antenna is excited by the use of 1 W power. However, it is imperative to note that the study of SAR is conducted here based on the optimal 1 W excited power regulated by the FCC. To ensure safety, decreasing excitation power at the Tx end is advisable, and the peak SAR value must be kept to the specified limit^17,23^. In the context of the introduced system, if the excitation power is decreased to 0.46 W at the Tx patch, the safety value of the SAR (1.6 W/kg) is achieved. Henceforth, by adjusting the excitation power at the Tx patch, the presented MTM-PC integrated system can be effectively utilized to power IMDs wirelessly.

**Fig. 17:**
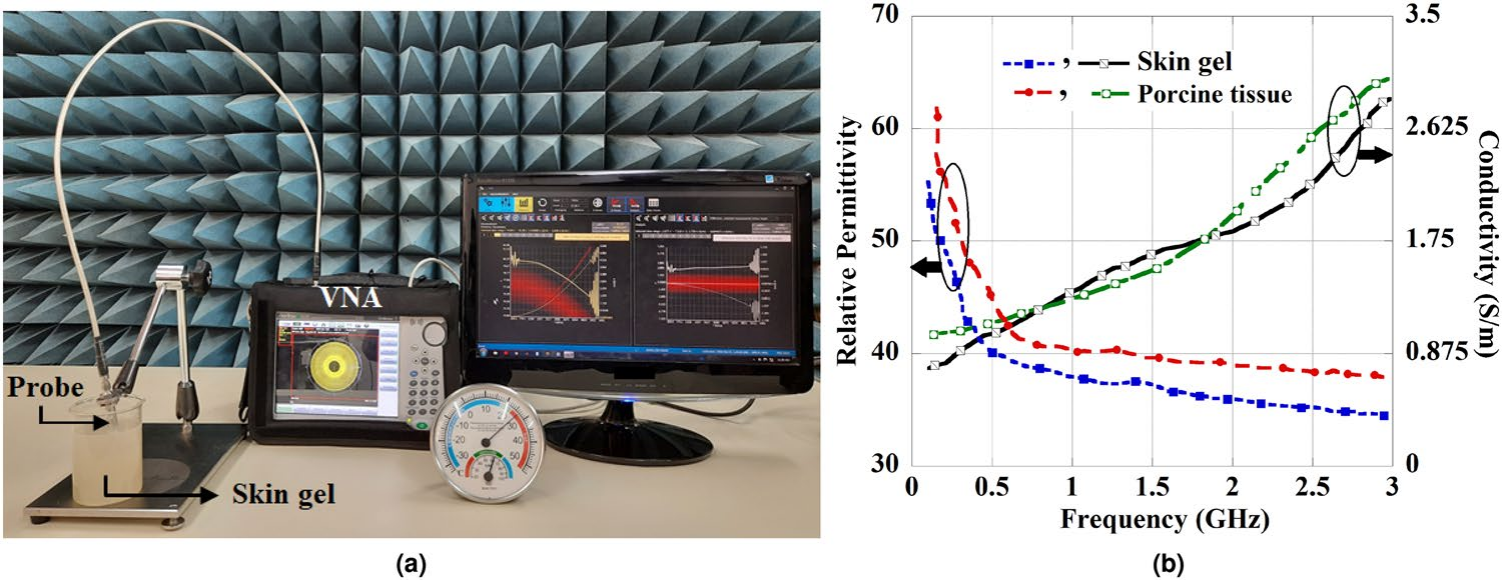
Measurement of the dielectric properties of the skin-mimicking gel and porcine tissue. (**a**) Measurement setup, and (**b**) obtained measured results.

**Fig. 18:**
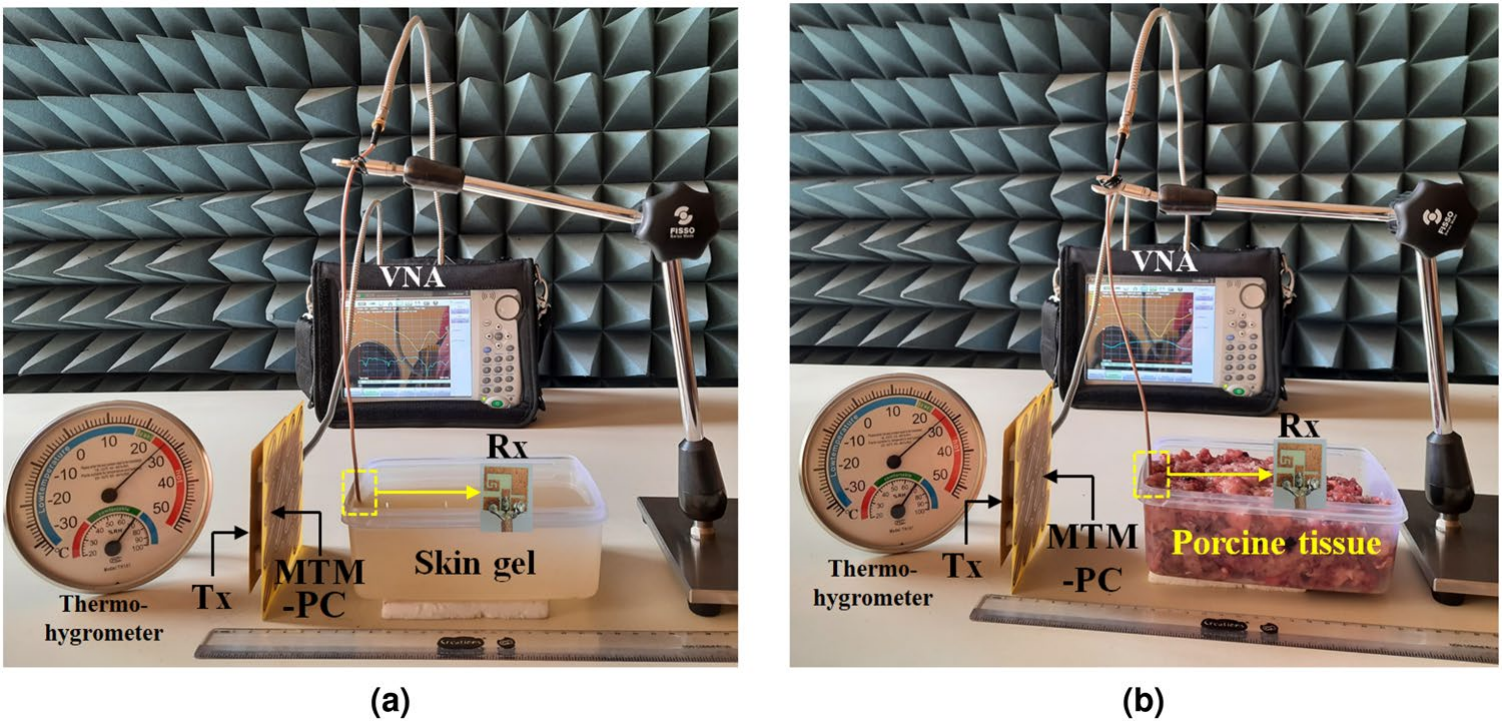
Experimental setup for MTM-PC integrated implantable WPT system. (**a**) Skin gel, and (**b**) porcine tissue.

## Measurement and discussion

In order to verify the introduced approach of the CP-antenna-based implant WPT system integrated with MTM-PC, prototypes of the Tx, Rx, and MTM-PC slab with $4\times 4$ unit cell array were fabricated. The fabricated prototypes are depicted in Fig. 15. To ensure the compatibility of the fabricated Rx implant slot with the human tissue and also to overcome the direct contact with tissue, a coating layer of alumina ($Al_2O_3$) having a thickness of 0.02 mm is applied. The process of coating the prototype of the Rx antenna with the $Al_2O_3$ layer has been thoroughly discussed in previous studies^23,27^. The optical perspective view of the alumina coating around the implantable slot antenna is important from design validation to performance optimization and ensuring bio-compatibility. Henceforth, the 3-D optical view of the alumina layer coated around the CP implant slot captured by a profilometer (Bruker Contour GT-K) is presented in Fig. 16. It can be observed from the figure that the coating has a nearly consistent thickness, which uniformly covers the implantable slot without irregularities.

To consider a practical environment, the measurements were carried out using homogeneous skin gel and porcine tissue. The ingredients for preparing the skin gel involve combining 47% deionized water, 53% sugar, and 1 gram of agarose powder for every 100 ml of solution^43^. The porcine tissue has been considered for measurements due to its dielectric properties being closely analogous to human skin tissue^44^. A vector network analyzer (VNA) and Speag Dielectric Assessment Kit-3.5 were utilized to measure the dielectric properties of both skin-mimicking gel and porcine tissue. Figure 17a represents the measurement setup, whereas Fig. 17b illustrates the measured electrical properties in the form of permittivity and conductivity of the skin gel and porcine tissue. The measurements are performed according to the guidelines discussed by DAK^45^. These measured results are much closer to the properties of the skin tissue model as used in the numerical analysis in the desired frequency range.

**Fig. 19:**
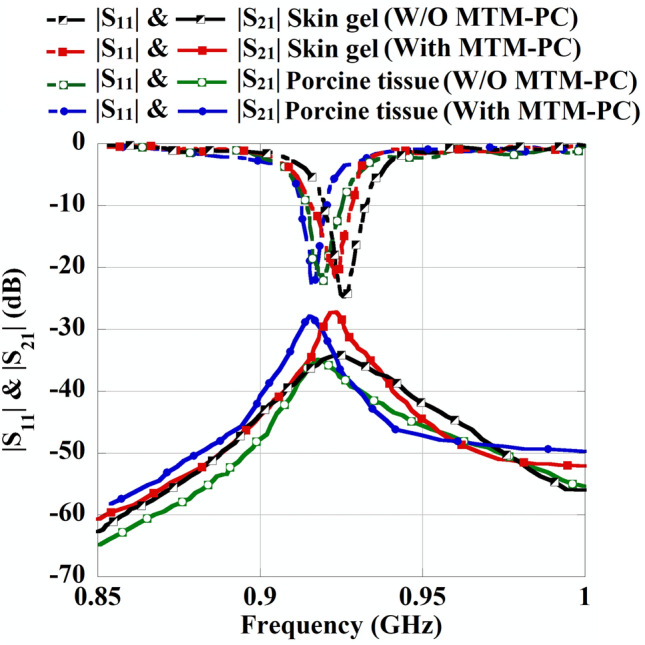
Measured S-parameter properties with and without MTM-PC integration.

The real-time laboratory measurement setup for the MTM-PC-based implantable WPT system is depicted in Fig. 18a and b for skin-mimicking gel and porcine tissue, respectively. The open-ended Rx slot is inserted at a depth of 4 mm in both the skin gel and porcine tissue. A 50 $\Omega$ coaxial cable with an SMA connector is employed to connect the Rx antenna. The Tx antenna is positioned at a distance of 50 mm from the skin layer. The MTM slab is mounted at 5 mm apart from the Tx patch using Styrofoam. To ensure precise experimental results, the Tx patch, MTM-PC slab, and CP implant are properly placed beside the identical horizontal line, as shown in Fig. 18.

**Table 1:** Comprehensive study among the simulated and measured results for both with and without (W/O) the inclusion of MTM-PC.

Results	MTM-PC loading conditions	Frequency (MHz)	$|S_{11}|$ (dB)	$|S_{21}|$ (dB)	Improvement (dB)
Skin model (Sim.)	W/O MTM-PC	922	$-$ 19.13	$-$ 32.58	–
	With MTM-PC	919	$-$ 21.37	$-$ 25.64	6.94
Skin gel (Meas.)	W/O MTM-PC	925	$-$ 24.36	$-$ 34.26	–
	With MTM-PC	923	$-$ 21.57	$-$ 27.22	7.04
Porcine tissue (Meas.)	W/O MTM-PC	917	$-$ 23.21	$-$ 34.62	–
	With MTM-PC	915	$-$ 23.10	$-$ 27.86	6.76

**Fig. 20:**
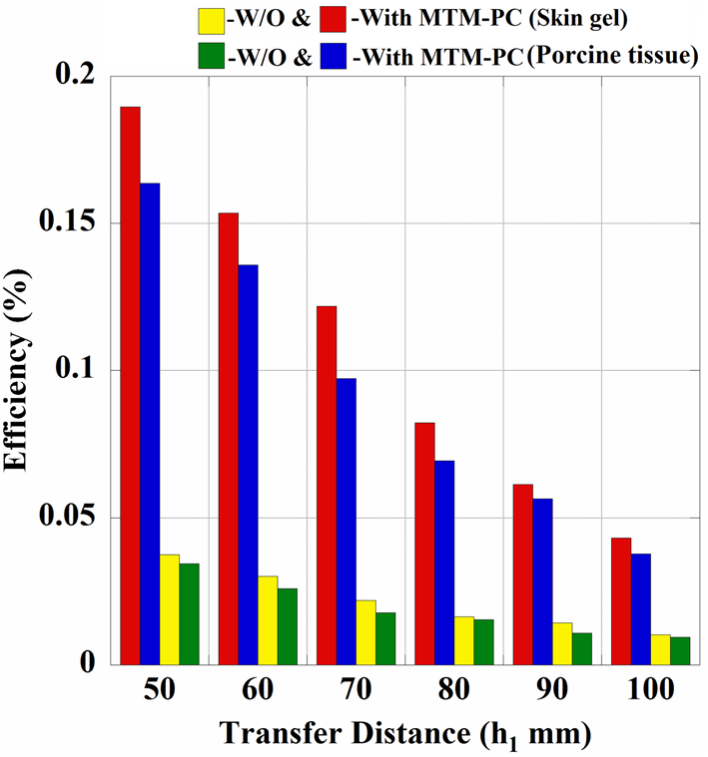
Change in power transfer efficiency with the distances ($h_1$).

**Table 2:** Comparative analysis between this work and the state-of-the-art work reported in the literature.

Refs.	Working frequency (GHz)	Volume of *Rx* implant (${\rm mm}^{3}$)	Via/Shorting-pin in *Rx* implant	Bending/bio-compatible of *Rx*	LP/CP WPT system	Implant epth (mm)	System’s operating field	Tx distance from tissue (mm)	$|S_{21}|$ (dB)/efficiency ($\%$)	Implant environment for simulation
^13^	0.430	152.40	No	No/No	LP	3	Near-field	60	– 27.9/0.16	Skin model
^17^	2.45	57.60	No	Yes/No	LP	3	Near-field	50	– 18.98/1.26	Skin model
^18^	0.430	91.44	No	No/No	LP	4.5	Near-field	50	- 27/0.19	Muscle model
^19^	2.45	6.56	Yes	No/Yes	LP	55	Far-field	60	– 18.6/1.38	Abdomen model
^23^	2.45	49.28	No	Yes/Yes	LP	3	Near-field	60	– 14.86/3.26	Skin model
^24^	0.433	40.22	No	No/No	LP	4	Near-field	10	– 6.91/20.3	Skin model
^25^	0.915	153.67	No	No/No	CP	4	Near-field	20	– 38.9/0.012	Skin model
^27^	2.45	34.68	No	Yes/Yes	CP	2	Near-field	40	– 29.74/0.11	Skin model
^28^	2.40	127	Yes	No/No	CP	3	Near-field	100	– 38.23/0.015	Skin model
This work	0.919 (Sim.)								– 25.64/0.27	Skin model
	0.923 (Meas.)	29.23	No	Yes/Yes	CP	4	Near-field	50	– 27.22/0.19	Skin gel
	0.915 (Meas.)								– 27.86/0.16	Porcine tissue

Furthermore, a comparison of the measured S-parameter properties of the introduced WPT system in skin gel and porcine tissue, for both with and without integration of MTM-PC is depicted in Fig. 19. A significant increment in the coupling strength of $|S_{21}|$ can be seen from the plot with the integration of the MTM-PC into the system, leading to an overall enhancement in PTE. A detailed study of the measured and simulated results in two distinct implant environments is elaborated in Table 1 at $h_1 = 50$ mm for a better understanding. The table shows that integrating the MTM-PC slab in the system results in an approximately 6.94 dB increase in the amplitude of $|S_{21}|$ from numerical simulation. Meanwhile, experimental results achieved from skin and porcine tissue indicate an improvement of 7.04 dB and 6.76 dB, respectively. Moreover, to evaluate the impact of MTM-PC on the efficiency of the proposed system, the distance ($h_1$) is increased while keeping the MTM-PC attached over the Tx antenna. The variation in measured PTE resulting from changes in the distance, for both with and without presence of the MTM-PC, is exhibited in Fig. 20. The system’s PTE is determined utilizing the maximum amplitude of $|S_{21}|$. Typically, an increase in transfer distance leads to a decrease in system efficiency. Nevertheless, the inclusion of the MTM-PC slab significantly enhances the PTE of the introduced system.

Finally, a detailed, comprehensive study of the introduced WPT system against the recent state-of-the-art work is conducted, and the outcomes are detailed in Table 2. Notably, our proposed implantable Rx antenna stands out by occupying the smallest volume compared to the reported works^13,17,18,23–25,27,28^. Additionally, the flexibility and bio-compatibility of the implantable Rx element with the human body are key features compared to the described works^13,18,24,25,28^. Moreover, our proposed system achieves better coupling strength ($|S_{21}|$), particularly compared to the CP-based reported literature^25,27,28^, leading to an enhanced and better PTE. The use of a CP-based WPT system in the proposed study eliminates the issues of mismatch loss and multi-path interference faced by LP-based WPT systems^13,17–19,23,24^. Furthermore, the proposed approach simplifies the design of the WPT system by employing a straightforward CP implantable antenna configuration, eliminating the use of vias or shorting pins^19,28^. Therefore, the proposed system not only reduces design complexity but also avoids additional losses due to the use of vias or shorting pins.

## Conclusion

This article discusses the development, evaluation, and practical testing of a rotation-insensitive, bio-compatible WPT system specifically designed for implantable medical devices (IMDs). The proposed system establishes a WPT link using a simple arrangement consisting of a CP-Rx implant antenna and a Tx patch antenna operating at the frequency of 920 MHz. Furthermore, in the proposed system, the unique HRI and PC property of an MTM slab has been utilized, leading to a significant enhancement in PTE compared to existing works. To ensure compatibility with the human tissue, a bio-compatible alumina layer is used to encapsulate the flexible Rx implant antenna. Notably, the proposed system demonstrates performance stability with the rotational effect of the Rx implant. A detailed numerical study of the SAR is conducted to confirm the feasibility of the proposed system in real-time application. Additionally, the CP-based implant WPT system with enhanced PTE, designed to handle rotation, along with the flexibility and bio-compatibility of the Rx implant, opens up promising features to charge the IMDs wirelessly.

## Data Availability

The datasets generated and analyzed during the current study are available from the corresponding author upon reasonable request.
